# Overseas Medical Students in Ukraine and War-Related Interruption in Education: Global Health Considerations from India

**DOI:** 10.5334/aogh.3926

**Published:** 2022-11-03

**Authors:** Shubhajeet Roy, Vivek Bhat, Ahmad Ozair

**Affiliations:** 1 Faculty of Medicine, King George’s Medical University, Lucknow, India; 2 St. John’s Medical College, Bangalore, India

**Keywords:** medical education, workforce development, Russian invasion, global health, public health

## Abstract

**Background:**

The Russian invasion of Ukraine in 2022 has caused a humanitarian crisis impacting millions of individuals within Ukraine and globally. While war-related healthcare delivery has been discussed in both the academic biomedical literature and in non-peer-reviewed sources, little academic attention has been paid to overseas medical students who have had to abandon their education. These constitute nearly a third of the 80,000 international students in Ukraine and represent a valuable part of the global healthcare workforce.

**Objective:**

This article utilizes the illustrative case of the over 18,000 Indian-origin medical students enrolled in Ukraine to review the state of overseas medical students in Ukraine and war-related interruption in education.

**Methods:**

Literature, both academic and non-academic, published up until October 1, 2022, pertaining to the conflict, the impact on students, the demands of various stakeholders, and the proposed solutions, was reviewed through the use of appropriate keyword-based searches.

**Findings:**

Factors influencing the decision to pursue education in Ukraine and their pre-crisis pathway to home practice are first discussed. Indian-origin students have historically gone on to Ukraine after securing insufficiently competitive ranks in the national medical school entrance exam, thus preventing them from getting a heavily subsidized education at publicly funded institutions in India. In the 2022 Russo-Ukrainian conflict, these students have faced not only actual and potential challenges related to their safety, shelter, food, and home return, they have been considerably impacted by the uncertainty regarding their educational prospects back home. The article then delineates the nuances and challenges in attempting to reintegrate a large potential healthcare workforce in the home country, and discusses possible solutions, especially those being implemented by the government.

**Conclusion:**

Urgent need exists for the involvement of all stakeholders and careful consensus-building before the proposed reintegration from an equity-based perspective.

## Introduction

The Russian invasion of Ukraine that began on 24^th^ February 2022 has impacted millions across the world. While conflict between the two countries had been occurring for almost a decade, this large-scale, unprecedented incursion has caused a humanitarian crisis. 20,000–50,000 casualties have been reported, and over five million residents of Ukraine have had to flee since the start of the conflict [1,2,3,4].

The war has had an overarching impact on populations worldwide, as evident in the global challenges related to the supply of oil/gasoline and cereal grains [1,2,3,4]. While all Ukrainian communities have suffered a large number of deaths, students in Ukraine in particular have additionally suffered a loss of future skill development and have had livelihood prospects impaired. The population-level impact of disturbed career prospects of a large cohort of future working professionals, including physicians-in-training, is likely sizable [5]. While war-related healthcare delivery has been discussed in both the academic biomedical literature and in non-peer-reviewed sources [6,7], little academic attention has been paid to the thousands of overseas medical students who have had to abandon their education in Ukraine. In particular, the complex nuances behind their pursuit of foreign medical education and the challenges of reintegration in their home countries remain undiscussed, even though some brief calls to help trainees have been published in the literature [6,7,8,9,10,11].

This article aims to utilize the illustrative case of the over 18,000 Indian-origin medical students enrolled in Ukraine to review the state of overseas medical students in Ukraine and war-related interruption in education. Literature, both academic and non-academic, published up until October 1, 2022, pertaining to the conflict, the impact on students, the demands of various stakeholders, and the proposed solutions, was reviewed through the use of appropriate keyword-based searches.

## Overseas Medical Students in Ukraine

The 2022 conflict, by disrupting medical education in Ukraine, has impacted healthcare workforce development, not only in the country but on a global scale. To recognize this, it is essential to note that a large number of overseas individuals, including from India, had historically been part of Ukraine’s populace [12]. Before the crisis, a Ukrainian Ministry of Education report had indicated that over 80,000 students from over 150 countries had been enrolled in Ukraine [5]. Of these, nearly half were in professional education related to healthcare, with over 26,000 students from overseas enrolled in Ukrainian medical schools. Many of these schools often had more than 50% of their class strength constituted by overseas students. In particular, estimates from India state that almost 18,000 Indians were enrolled in medical schools across Ukraine before the war [13,14]. These students were among the first to be affected by the invasion since several Ukrainian medical schools with Indian students were in the eastern part of Ukraine, which was the first region to be invaded by Russia [15]. Similarly affected were the thousands of Pakistani-origin students enrolled with them as well [16].

The multidimensional impact on these students carries implications for the healthcare workforce, not just in South Asia where most of these physicians-in-training return to practice, but across the world. For instance, in the United States (US), a 2021 report by the Federation of State Medical Boards (FSMB) indicated that international medical graduates (IMGs) constitute over a fifth of all licensed US physicians [17]. Similarly, the General Medical Council (GMC) of the United Kingdom (UK) reported that more individuals (IMGs) from outside the UK and Europe joined the UK medical workforce in 2020 than those from the UK and Europe combined [18].

The majority of these overseas students have been evacuated by their respective governments, with India having had a particularly high rate of successful evacuations [19,20]. However, given that they cannot continue their medical education in Ukraine for the foreseeable future, significant concerns remain. Discussed below is the impact thereof and challenges behind the educational reintegration of overseas candidates of Ukrainian medical education, in particular from India. As of October 1, 2022, relevant national administrative organizations in India have not decided favorably regarding their reintegration into Indian medical schools. Hence, we also highlight potential solutions to the challenge of reintegration.

## Medical Education in Ukraine

A comprehensive understanding of why there are over 18,000 Indian students enrolled in Ukrainian medical schools may be obtained by examination of the pre-crisis state of medical education in Ukraine and India. Recognition of this historical context is also critical in appreciating the nuances of and the potential challenges against reintegration, particularly from financial and ethical perspectives.

Medical school in Ukraine begins straight after high school, similar to the arrangement in India, Pakistan, Bangladesh, and Sri Lanka, along with the majority of spots in the UK and Europe, amongst others. This is a five-and-a-half-year course, after which the students are awarded the Bachelor of Medicine & Bachelor of Surgery (MBBS) degree. The course includes one year of a compulsory rotatory internship, wherein MBBS candidates work in major clinical departments with a provisional medical license, but under senior supervision and with a limited scope of practice.

India’s National Medical Commission (NMC), the erstwhile Medical Council of India (MCI), currently recognizes MBBS degrees from 45 medical schools in Ukraine for practice in India. For these foreign medical graduates (FMGs) to obtain licensure to practice, they must complete their internship in that foreign country and then clear the MCI Screening Test. This criterion-referenced test, now widely known as the Foreign Medical Graduates Examination (FMGE), was started in 2002 and has historically had dismal pass rates [21,22]. Held biannually, it is anecdotally known that those failing to clear it attempt it several times. After passing FMGE, FMGs may either practice as general physicians or attempt to get selected for residency training in India.

## Medical Education in India

Given the rapidly evolving nature of events, the terminology related to medical school admissions is described below. The country has over 550 NMC-accredited medical schools which offer over 80,000 positions annually for a five-and-a-half-year-long undergraduate MBBS program. Unlike several other countries that utilize a holistic application system, such as the US, UK, Canada, and Australia, the Indian system continues to depend only on an annually held, standardized, norm-referenced test.

Admission to medical school is thus based solely on the test-day performance on a single examination, called the National Entrance cum Eligibility Test – Undergraduates (NEET-UG). Candidates obtaining a score higher than a set threshold, which is determined by the National Testing Agency (NTA) of the Government of India, are said to have ‘qualified’ NEET-UG. This, by no means, is a guarantee of a position, as over 50% of the test-takers qualify [23]. It merely indicates that the candidate is suitable for proceeding with medical education either in India or abroad since all students going overseas must necessarily ‘qualify’ NEET-UG [24]. In addition to their score, each examinee receives an all-India rank (AIR), as well as a ‘category rank’.

Private medical schools are permitted to offer all their seats to ‘general category’ candidates, that is, they are permitted to not have affirmative action policies. Meanwhile, the system offers, in the case of publicly funded schools, nearly half the spots for the general category, and reserves the other half for candidates from historically marginalized groups. Major reserved categories include Scheduled Castes (SC), having 15% of these spots reserved, Scheduled Tribes (ST) having 7.5% spots reserved, and Other-Backward Classes belonging to Non-Creamy Layer (OBC-NCL), having 27% reservation [25]. This represents a formal fulfillment of an affirmative action policy, given that these groups are considerably under-represented in medicine. This system of reservation has also contributed to considerable hostility amidst candidates not belonging to reserved categories, a fact that is of consideration in the overseas education issue [26].

Post-examination, multiple rounds of a process called ‘counseling’ occur wherein the candidate digitally submits a rank order list of preferred schools and the system allots their best possible choice of the institution based on their AIR, category rank, and the number of positions remaining – the process repeating several times until all spots have been filled. Thus, the first ranker has access to all spots in the country, while somebody having an AIR of 10,000, would expect to obtain a spot that may not be at the top of their list, more so if they belong to the general category. Notably, the competitiveness and the processes associated with NEET-UG are quite similar to the annually held exam used for residency selection, known as the NEET-Post-Graduate (NEET-PG).

Two financial tiers of undergraduate medical education exist in India, one having heavily subsidized spots, and the other having fully self-funded ones, with some differences in quality and extreme differences in cost between the two. Typically, the subsidized education is through (1) *all* spots at publicly funded medical schools, more commonly known in India as Government Medical Colleges (GMCs), or (2) *some* spots in select private institutions where the provincial government has asked for earmarking of subsidized seats. The remaining seats, typically always in private institutions, require the candidate to submit typically a few orders of magnitude higher tuition fees than subsidized spots. This is further compounded by publicly funded medical schools having a generally higher quality of clinical training [27], as reflected in both national hospital rankings published annually by the Government of India [28] and in medical school rankings of external agencies [29]. For instance, one major private ranking system ranked only publicly funded schools as the top ten in India [30].

## Factors Driving Pursuit of Overseas Medical Education in Ukraine

Popular destinations for Indian students who move abroad for medical training include Russia, Ukraine, the Philippines, Germany, Kazakhstan, and China [31]. Among these, several factors have come together to make Ukraine a highly preferred destination for Indian students, with the chief factors being (1) a mismatch between the number of applicants and the number of subsidized medical school seats in India, (2) the cost of non-subsidized medical education in India, compared to Ukraine, and (3) quality and recognition of education in Ukraine compared with other overseas alternatives.

### (1) Supply-Demand Mismatch

The primary factor which drives students to pursue medical education is the extremely competitive nature of medical school admissions in India. In 2021, over 1.6 million candidates registered for the NEET-UG, while the total number of available medical school spots was 83,075, resulting in a supply-demand mismatch of 20 times [32]. Of these spots, those at publicly funded schools are more competitive, stemming not just from the better clinical education, but also their affordability compared to privately funded schools.

This mismatch is supposedly worsened for ‘general category’ candidates because half the seats in government-funded medical schools are reserved for certain communities [33]. As further described below, private schools in India frequently cost more than foreign medical schools. Hence, many candidates, particularly from lower- and lower-middle-class families seek opportunities abroad if unable to secure a spot in India.

Meanwhile, admission to Ukrainian medical schools has historically been far less competitive. Their self-stated primary admission requirement has typically been that candidates must have scored at least 50% in their high school final examinations. that is, the ‘10+2 board examination’ in the home country, with no additional test-taking required.

### (2) Lower Attendance Costs at Ukrainian Medical Schools

Tuition fees in Indian privately funded schools can be as high as 13.3 million INR (almost 180,000 USD) for the entire course – an expenditure that is typically out of reach for the average Indian aspirant [9]. Meanwhile, tuition fees in most Ukrainian medical schools are known to be much more affordable, costing around 30,000 USD for the entire course [34,35]. Additionally, attendance costs in Ukraine are lower than in most other popular foreign destinations for Indian medical students. For example, in Russia, Kyrgyzstan, and Poland, attendance costs for the entire MBBS course are approximately around 41,000, 36,000, and 72,000 USD respectively. Finally, the cost of living in Ukraine is comparatively low, travel to Ukraine is relatively cheap, and travel within Ukraine is subsidized for medical students [35,36].

### (3) Quality and Recognition of Ukrainian Education over Alternatives

Several factors related to the quality and the international recognition of the coursework have made Ukraine a more attractive choice. First, in most of the popular locations for overseas medical education, the country’s national language is the typical medium of instruction. For example, in China, as of 2021, only 45 medical schools out of over 420 had English as the primary medium of instruction. Similarly, in Russia, only 25 out of 70 medical schools utilize English instruction [37,38]. In Ukraine, however, English has uniformly been the medium of instruction.

Second, for Indian students who may wish to return to their home country, trends in the biannually held FMGE are a major factor of consideration. The national pass rates of graduates from Ukraine are higher than those of other countries. Official data spanning from 2015–2018 report that 16% of Ukrainian graduates passed the FMGE, compared to a global pass rate of 14%, and pass rates of 12% and 13% among graduates from China and Russia respectively [39]. Further, recognizing the needs of these Indian students, numerous private institutes have opened in Ukraine offering classes for the FMGE. Pre-crisis, the Ukrainian Government, through its official reports, had also indicated support for the home return of overseas candidates [5]. Notably, no academic literature exists regarding the outcomes of those failing the FMGE, especially concerning the mean number of attempts required, how many FMGs finally practice in India, and how many emigrate.

Third, MBBS degrees awarded in Ukraine are recognized in India, the US, Canada, the UK, and most countries in Europe, ensuring that candidates can return home or go elsewhere for further training or general practice. The vast majority of Ukrainian medical schools are listed in the World Directory of Medical Schools (WDOMS) – a database maintained by the World Federation for Medical Education (WFME) and the Foundation for Advancement of International Medical Education and Research (FAIMER) [40]. Thus, these schools are recognized by the Educational Commission for Foreign Medical Graduates (ECFMG), an organization headquartered in the United States (US), but responsible for primary source verification (PSV) for medical regulators in the US, Canada, UK, Australia, and so on. For comparison, at the time of writing, from China, only 162 medical schools, out of over 420, are listed as operational on WDOMS [41]. However, the authors of this work do note that the WDOMS/WFME accreditation process is now changing – it is moving from the listing of individual schools in different countries to the accreditation of the national body overseeing schools in the country [40]. Further, Ukraine is part of the European Higher Education Area (EHEA), and follows the Bologna process for the standardization of European higher education, unlike China, Russia, and the Philippines, for instance [42,43,44]. Additionally, Ukrainian medical school degrees are recognized by the UK’s General Medical Council. This means that graduates have the opportunities available for training and practice in the high-income countries of North America or Western Europe, should they wish to do so.

## War-Related Impact on Returning Medical Students

Prior studies from medical students in other war-torn countries provide some indications regarding the possible current mental status of returned students. These studies reported heightened levels of anxiety, depression, and suicidal ideation in their participants [45]. Besides fearing for their well-being, shelter, food, and transport, affected students reported significant uncertainty regarding their clinical competence and career prospects, with this uncertainty being a contributory factor to poor mental health [46,47]. Similar parallels may be drawn regarding the students from Ukraine. Likely, the destruction of their second home and the deaths of its people have had a significant impact on the students that have safely escaped. All this comes in the background of the COVID-19 pandemic, where medical students particularly have already had their education compromised, and education-related stress heightened [48,49].

Continued education in Ukrainian medical schools is uncertain, with a low likelihood of return for these overseas students. Some schools have resumed online classes for these students and declared that they will hold exams later in the year, in safer parts of the country [50]. However, detailed instructions are sparse.

For students who wish to continue their education in India, their prospects are just as unclear. Individuals at different stages of their six-year medical school program face different challenges. Candidates early in their medical training may be concerned about the prospect of preparing for NEET-UG once again, having already spent significant time and effort to obtain a medical training spot abroad. Preparing for this exam, which is based on high school subjects and requires focused preparation for several years, will be more difficult with each year of medical training completed. Meanwhile, senior medical students may face concerns about whether their years of training, almost to completion, will need to be repeated. Notably, FMGs have historically had to complete the internship year in the overseas country for becoming eligible to take the FMGE. Without being eligible to take the FMGE, an exam for which there has never been a mass exemption granted, overseas students may potentially never be able to practice in India.

## Protests and Demands of Returning Students

Since their return to India, returning students have struggled, initially with uncertain directions from authorities, and later, with an outright refusal of support from national organizations. After their return in February-March 2022, aggrieved students filed several public interest litigations (PILs), that is, ‘litigations undertaken to secure public interest and demonstrate the availability of justice to socially disadvantaged parties’, in the Indian Supreme Court, as well as the New Delhi High Court seeking clarifications regarding their continued education. Their plea to the Supreme Court also sought for the Indian government to provide an orientation program for potential admission to Indian medical schools [51]. The Indian Medical Association (IMA), the pan-national body of Indian physicians, wrote a formal letter to the Prime Minister in support of these students [52].

Primarily, these students have demanded reintegration into publicly funded Indian medical schools through direct lateral entry, the same ones described above as being the most competitive institutions for obtaining admissions through the NEET-UG. On May 15, 2022, overseas students hailing from the state of Tamil Nadu, staged a large protest in the state’s capital Chennai, requesting the state and Central governments to look into their concerns [53]. Similarly, on May 17, 2022, a group of these students from the state of Uttar Pradesh (UP), met the state’s Chief Minister, asking for a continuation of their education in any of the state medical schools [54]. Additionally, parents of overseas medical students formed a body to have their demands better heard, named the ‘Parents’ Association of Ukraine MBBS Students’. The latter held a major press conference on June 23, 2022, expressing their concerns about their children’s academic losses, and seeking urgent federal intervention to accommodate their children at the local medical colleges as a one-time measure [55]. On June 25, 2022, a large group of students and their parents from multiple states, staged a demonstration outside the NMC office in New Delhi, the country’s capital, reiterating their demands [56]. June 26, 2022, saw another group of students start a hunger strike in the nation’s capital, in the hopes of prompting urgent national action [57].

## Multi-Dimensional Challenges to Reintegration

Significant challenges hinder the reintegration of overseas medical students into established Indian medical schools. In addition to ethical challenges to equity and justice, there are numerous logistical barriers to potential solutions, including but not limited to the admissions process, medical school capacity, and academic overburden on clinical faculty. Importantly, the Government of India has no legal obligation to ensure the completion of these students’ medical training, even though arguments may be made regarding a moral obligation [58]. Furthermore, while these individuals may be seen as a potentially useful future healthcare workforce, their current status as students requires a considerable investment of public and private resources, thus preventing the enforcement of a moral obligation for non-home countries to help in the reintegration process.

The decision to reintegrate carries ethical concerns of equity and justice. It must be understood that while competition is significant for any publicly funded medical school spot in India, it is vastly more intense for the top-ranked colleges. It is widely known across the country that pre-medical students in India, who have secured sufficiently high ranks in the NEET-UG, had historically begun preparations for the exam at the start of high school at the latest and frequently even earlier. Given the pre-existing supply-demand mismatch, there exist several individuals who, because of their rank, had to opt for an institution that they did not originally want, because that was the spot available to them, or had to join private schools at significant cost. For these individuals, who obtained better ranks than the Ukrainian students and are studying in India, the reintegration of lower-ranked overseas candidates into schools ranked better than their current ones would be unjust and would go against the principle of equity. This concern is further aggravated when one considers the case of low-income Indian high school students. Often, students from resource-limited families see the publicly funded medical school spots as their primary source of upwards mobility, given that they would be unable to pay the fees for education either in private institutions in India or in Ukraine. While relevant academic literature is limited, it is widely known in India that there is not an insignificant number of contemporary students who could not obtain a publicly funded medical school spot and had to take a year off to prepare again for the annual examination. Thus, permitting the returned students to join local medical schools would potentially be a violation of the merit-based system that is the fundamental basis of NEET-UG.

Consider the complex scenario where these students are permitted to enter Indian medical education. With over 550 medical schools in India, it is hypothetically possible to accommodate the vast majority of these students. Historically, ranks of medical schools have driven applicant choice, with the schools themselves lacking any say in selecting candidates. The top rankers in the NEET-UG uniformly choose the top-ranked colleges [59]. Thus, these students’ demand for publicly funded medical spots has an unclear basis, given that most students studying in Ukraine had not secured a high enough rank to secure a publicly funded spot, driving their pursuit of overseas education [60,61]. This point is best highlighted by the case of allotting a Ukrainian return student an additional spot at the All India Institute of Medical Sciences (AIIMS), New Delhi. For a general category student to gain admission to the historically consistent number one medical school choice opted for after NEET-UG, they must typically score among the top 100 students in the >1.6 million test takers annually. Those obtaining admission to AIIMS New Delhi, receive cultural adulation in a manner, akin to hero worship, that may be challenging to fully comprehend for non-Indian residents [62,63,64,65,66,67]. Thus, the allotment of such a prized spot to a Ukrainian may lead to considerable discontentment amongst previous test-takers, and may likely lead to widespread protests.

Additionally, several logistical questions come to the fore. Should the students be assigned only publicly funded schools, given that they are under the purview of the government; or should privately-funded schools also be asked to help with the reintegration? What determines the number of students allotted to each Indian institution – should it be based on the existing number of approved seats, for instance, a 20% additional capacity for accommodating Ukrainian students per institution? Should medical schools themselves have a say in this new allotment? Given the intensely ranked-choice nature of medical school selection in India, what factor basis should drive the assignment of overseas students to Indian schools? Given the differences between Indian and Ukrainian curricula, how much credit should be transferred? Consider, for instance, third-year Ukrainian medical students requesting the maximal amount of their education be considered for credit back – two years – are those two years of Ukrainian education sufficient for Indian standards, given the new vertically integrated NMC 2019 curricula? Such questions remain unclear.

Finally, questions regarding the baseline and the final competency of these candidates have been raised, the former being more justified than the latter. With regards to their baseline competency, these candidates typically scored much lower on the NEET-UG than their counterparts training in medical schools in India, forcing them to move to foreign medical schools [68,69]. However, given the lack of literature, it is unclear as to what level of baseline competency in NEET-UG is an appropriate predictor, if at all, of being a safe and competent physician. With regards to competency after medical school, the dismal pass percentages in the FMGE, typically between 10–20% overall per year, have raised warranted questions about the quality of medical training in foreign schools [39,68]. While these questions regarding the possession of required competencies by graduating students are justified, it should also be considered that medical graduates from India currently do not give any sort of exit exam. It may well be possible that not dissimilar pass rates may be seen for Indian medical school graduates upon being mandated to take the same exit exam. Notably, a national exit exam (NEXT) is planned for the future, which is currently planned as an annually held test to be taken by all Indian and foreign graduates, whose passage will provide a license to practice and scores on which will provide ranks for residency selection [70,71]. Thus, the purposes served by FMGE and NEET-PG for foreign graduates will be served together by NEXT in the future.

## Potential Benefits of Reintegration

Despite these barriers, these students are Indian citizens and physicians-in-training, many hoping to contribute to the country’s overburdened healthcare system. The country has a significant deficit of trained healthcare workers, having far fewer physicians than the World Health Organization’s (WHO) recommendation of 10 doctors per 10,000 people [70]. While estimates vary, an analysis of labor databases managed by the National Sample Survey Office (NSSO) of the Government of India, had reported that the number of active, adequately qualified physicians was only 5 per 10,000 people in 2018 [72]. Data published in 2019 by the WHO itself indicated a ratio of 7.4 physicians per 10,000 people [71].

Rural areas bear the brunt of physician shortage due to heterogeneous distribution and clustering. Physicians typically prefer to practice in urban areas, with commonly cited reasons being better infrastructure along with greater opportunities for career progression and family support in these areas [73]. In 2015, it was reported that almost 10% of primary healthcare centers – the first point of contact between village residents and a physician – did not have a single doctor. In community health centers (CHCs), the next tier of rural healthcare, the situation was even direr. CHCs are officially recommended to have one physician, one surgeon, one pediatrician, and one gynecologist, yet in 2015, there were shortages of around 80% for each specialist [74,75]. Unfortunately, projections for the future have predicted little improvement. The analysis of NSSO data projected that the density of skilled healthcare workers would remain largely unchanged in 2030, while a slightly older analysis of the MCI’s database of registered doctors projected that India would have around 1 million active physicians in 2030, compared to a requirement of 1.5 million physicians [72,73,76].

From an ethical perspective, it may be argued that India strongly needs a much larger physician workforce, but provides limited training opportunities, leading to aspirants seeking the best possible alternatives. The country also suffers from considerable emigration, with a 2017 analysis reporting that over 90,000 Indian medical graduates were in active practice overseas [76].

From a financial standpoint, allowing these students’ training to be passively wasted would be a significant loss, given that state and federal governments in India spend a significant amount per year of publicly funded medical education [77,78]. The total amount for educating an MBBS student at AIIMS was estimated to be close to 17 million INR, as per a formal estimation using traditional costing methodology, that was published in 2013. Data collection for this estimate was carried out over thirteen years ago, so the cost has likely increased considerably since then [79]. Thus, the Indian government could save a significant amount by building on the partial training obtained by students in Ukraine. Graduating these students rapidly for entering into residency training may potentially provide India’s healthcare system with a vital injection of manpower and also add considerable economic value.

One potential nuance to this issue is having reintegration at the juncture of preclinical and clinical education in medical school. Compared to clinical training, preclinical education in medical schools across countries is more standardized, has curricular similarities, and shares common reference textbooks. While courses across countries may be structured differently, some disciplines – anatomy, physiology, pathophysiology, pharmacology, biochemistry, microbiology, immunology, genetics, molecular biology, and neuroscience – typically form the core of preclinical education worldwide. Meanwhile, undergraduate clinical education, given its focus on learning patient-centered care, is more tailored to local requirements and curricula. Most clinical subjects require students to have mastered communication in the predominant language of the patients coming to the affiliated hospital of their medical school, even though the medium of instruction may be different. Thus, assessing competency in preclinical subjects and thereafter transferring requisite credits may be done more reliably. In India, the rigid curriculum set by the NMC mandates the primary teaching of preclinical subjects must occur in the first two years, with minimal curricular flexibility permitted to individual medical schools, allowing for high levels of standardization. Thus, returning students who have completed their preclinical education in Ukraine may be reliably given transfer credits for two years of medical school in India.

While no formal economic analyses regarding the benefit of reintegration in this case have been published, we provide below a brief estimation that is based on several major assumptions. Let us assume that of the 18,000 Indian medical students in Ukraine, nearly 80% (N = 15,000) are rescued safely, a figure more conservative than official estimates, and wish to continue to continue their education and all of them apply for formal reintegration into government medical schools. Further, assuming that these students are uniformly distributed through different years of medical school, the current median education of a current student comes out to be year three. Given that the student has completed competency-related exams for two years, it may be reasonable to provide two years of transfer credits. Thus, in total, India will gain 2*15,000 person-years of completed training. If the traditional costing data from AIIMS of 3,131,000 INR per student per year is used, an expected total savings of 93,930,000,000 INR (93.93 billion INR or 1.15 billion USD) may be potentially made. While this number may be adjusted downwards given that educating a student at the most competitive medical school in India is likely to be amongst the costliest, consideration could also be made for adjusting upwards inflation, since data for the costing was reportedly collected during 2007–08 [79]. Unfortunately, we must acknowledge one glaring limitation to this estimate – to the best of our knowledge and literature search, there exist no newer Indian data regarding costs, be it from AIIMS or other medical schools. Usage of the AIIMS-estimated cost per student per year requires a high level of caution, given that its reporting article has major limitations and non-financial conflicts of interest – two of its authors were the founding editors-in-chief of the same journal, of which it was the first research article in the journal’s first published volume [79].

Nevertheless, while our figures are highly preliminary, these data do warrant grounds-level formal costing by the NMC and/or the Ministry of Health and Family Welfare (MoHFW), given that no national data could be found. A grounds-level costing may not only help the federal government estimate the public benefit of reintegration, but it may also help them in notifying all public medical schools to charge overseas returning students the same (or higher) annual fees that the costing provides. Meanwhile, the standard students enrolled through routine NEET-UG admissions would be charged the routine, highly subsidized fees. In this way, the medical education of these students remains self-funded, initially for two years in Ukraine and later in India. Thus, it would potentially not burden the economy, while helping add to the country’s healthcare workforce.

## Current State of Government Support for Reintegration

Currently, there exists little formal support for reintegration in Indian medical schools, particularly amongst the national authorities, despite several large-scale protest marches [80,81]. While no academic literature exists on this evolving subject, potential reasons behind the denial of the demand for reintegration are manifold, including those stated by the Government itself, as described below along with the evolving response of national authorities.

The Union Minister of State for the Ministry of Health and Family Welfare (MoHFW), Government of India had declared on July 22, 2022, that there were no legal provisions to accommodate these students in Indian medical schools. The National Medical Commission (NMC) had a similar stance of opposition to either accommodation in India or transfer elsewhere [80,81]. However, on August 3, 2022, the Committee on External Affairs (2021–22), in a report to the Indian Parliament, stated that the Ministry of External Affairs had recommended the MoHFW to allow these students to be enrolled in private Indian medical schools on a one-time exemption basis. As per the report, the committee believed that this was the only solution to the problem of these students and the only way they can complete their courses [80,81].

The Supreme Court, starting on August 26, 2022, then heard the case of seven petitions by affected students, who requested an appropriate solution given the recommendation of the Ministry of External Affairs. Here, the Court initially commented that these students were ‘not meritorious students in India’. In response to opposition from the students’ representative, who stated that these students had indeed cleared the NEET-UG, but could not afford privately funded medical schools, the Court retorted that regardless of their actual merit, these students had voluntarily chosen to go to Ukraine. This notwithstanding, their representative pointed out that the current situation was an extraordinary one and sought for the Supreme Court to direct the Central Government to act on the Ministry’s recommendation. He argued that the Government could exercise extraordinary powers under Section 45 of the NMC Act 2019 and direct the NMC to make an exception for these students. Additionally, the bench was made aware of the fact that students from other foreign countries, who had completed their MBBS studies, but not their internship due to the pandemic, had been permitted by the NMC to do the same in India. While Ukraine-returned interns had been given the same exception, provided they clear the FMGE, this would not apply to all the Ukraine returnees, as many of them had not reached internship yet [82,83,84]. The hearing was concluded by issuing notice on September 5, 2022.

The Solicitor General of India, representing the NMC, requested the Supreme Court for adjournment, in order for the NMC to deliberate [84]. The court agreed to adjourn the case till September 15. 2022. At this juncture, the NMC, in a reversal from its opposition [81], released a statement that it had no objection to any ‘academic mobility programs’ (AMP), that is, programs implemented privately, that allowed students to continue their education in countries other than Ukraine or India. However, the NMC clarified that the final MBBS degree must be awarded only by the original Ukrainian university and that these students were still required to pass the FMGE if they wished to return to India [85,86,87]. Meanwhile, several foreign medical schools, sensing an opportunity through the AMP, hiked fees from a maximum of 5000 USD/semester to 7000 USD/semester [85,86,87].

On September 15, 2022, the Central Government informed the Supreme Court that these students cannot be accommodated in Indian medical schools due to the absence of any such provision in the NMC act. Here, they stated their opposition to reintegration in India on the following grounds: (A) measures allowing such students might hamper the standard of medical education in India (B) these students went to Ukraine for two main reasons – low scores in NEET and affordability. Allowing students with poor merit in premier medical colleges in India could lead to further litigation. (C) These students wouldn’t be able to afford the fee structure in these private Indian colleges. Additionally, the Central Government, along with the NMC, professed their support for the official implementation of an AMP. However, the petitioners objected that there were not enough spots available in this program to accommodate all of them [85,86,87]. In response to this, the very next day, on September 16, the Supreme Court asked the Central Government to consider creating a web portal with details of foreign medical schools, where Ukraine-returned students could apply to complete their courses under the approved AMP [89]. On September 22, the NMC declared three medical schools in Georgia approved under the AMP [87], and on September 23, the Central Government officially informed the Supreme Court that the concerned federal organizations were working on creating the portal as requested [90]. However, they clarified that this program could not be used as a ‘backdoor entry’ to Indian colleges [88].

Along with the three major reasons stated by the Central government in its affidavit opposing reintegration, another major barrier to federal support is the likely fallout expected amongst large parts of the national populace if reintegration into publicly funded institutions is done. The NEET-UG and/or its predecessor (called AIPMT) have held historically high levels of socio-cultural significance [62,63,64,65,66,67]. NEET-UG is widely perceived as an engine of upward mobility and a pathway to generational success, similar to China’s National College Entrance Examination (NCEE), GaoKao [91,92,93,94]. The NEET-UG is administered by the National Testing Agency (NTA) of the Indian government itself, unlike most standardized tests worldwide such as MCAT, SAT, ACT, GRE, TOEFL, and OET, which are administered by private organizations. Notably, concerns have been raised regarding the supposed ‘non-profit’ classification of these administering organizations, which grants them tax deductions, but also permits them to charge significant fees for the exam and related preparation material [95,96,97,98]. NEET-UG must strictly adhere to curricula and textbooks developed by the National Council of Educational Research and Training (NCERT), an organization under the Government of India. All core textbooks, the material of which NEET-UG questions have to be framed material, are freely available online, while their hard copies are sold without profit to the government [99,100]. All of this has served to democratize access to and preparation for NEET-UG, for which students start preparing typically from grade 11 (two years of preparation), less commonly from grade 8 (four years of preparation), and rarely in grade 6 (six years of preparation) [94,9]. Affordable offline and online learning programs exist, including free classes on YouTube, where students learn material beyond their current grades in order to become ready for the NEET-UG, a praxis also performed by all authors of this review. A culture has thus long existed where NEET-UG aspirants and their parents believe that years of hard work will prove to be the deciding factor in scoring well and thus putting them on an upward trajectory, similar to the ‘Gaokao’ [62,63,64,65,66,67,91,92,93,94]. An attempt at reintegration is likely to be perceived to be against the decades of meritocracy that has been espoused culturally, and may incite mass public unrest. Therefore, the NMC and the Central Government have been hostile to the idea of reintegration, and no permissions have been granted at a national level from either of the legislative, the executive or the judiciary. In response, student protests across the country have continued.

Some state governments have extended support to these students, while the Supreme Court deliberates on the issue [14,17]. The government of the state of West Bengal permitted 412 students hailing from the state to continue training in the state’s publicly funded colleges. While initially appointed as observers, they were later allowed to attend lectures and practical classes. Of these, 78 students who had taken the same year’s NEET-UG were allowed to undergo counseling for ‘management quota seats’, typically the most expensive spots in private medical schools [101]. In March 2022, the health minister of the state of Karnataka had announced that around 700 returned medical students would be accommodated in around 60 different medical schools in the state. However, this announcement has not come to fruition. Similarly, in July 2022, the Chief Minister of the state of Tamil Nadu reiterated the state’s support for these students and requested that they be accommodated, in response to the central government’s statement to the Supreme Court [102].

## Possible Alternatives

There exists a need to examine all possible solutions to this issue and achieve consensus in consultation with several stakeholders, given that ‘direct reintegration into publicly funded medical schools’ represents a highly contentious option. Compared to this solution where consensus would be difficult, these alternatives may carry fewer socio-cultural and/or legal barriers, which may lead to potentially faster implementation. This is important given that these students were in the midst of their education, and the break in education may impact their future competency as physicians, given that interruptions in in-person clinical education from the now-receding COVID-19 pandemic still linger [103]. Additionally, if the pathway chosen is contentious, it may lead to further delays secondary to legal disputes, appeals, challenges in higher courts, retrials, appointments of task forces, and the like. Amidst this, the less privileged returning students will most suffer, given that inequity and disparities in medical education in India continue to rise [104].

One option, which is well-supported by current Indian medical students, is the retaking of NEET-UG in the next cycle by concerned candidates. While it fully complies with existing admission guidelines of NMC, it does raise questions of deliberate waste of resources and harsh decision-making for candidates afflicted by extraordinary events (*force majeure*). It is being estimated, informally, that nearly a third of returning students from Ukraine did take the NEET-UG [105]. Other than students in their internship year in Ukraine, students in all other years have been concerned about the prospect of performing well in the NEET-UG, having already spent significant time, money, and effort in obtaining medical education overseas. Preparing for NEET-UG, which requires standardized test-taking practice along with knowledge of physics, chemistry and biology, will be more difficult with more time invested in medical education. However, it does satisfy the harsh criteria of norm-referenced testing and meritocracy in a country granting these attributes socio-cultural sanctity [62,63,64,65,66,67], notwithstanding their inherent limitations.

Another potential solution is the transfer of these candidates to other foreign medical schools, with governmental and/or non-government-organizational support, as evident in the AMP. This avenue has been utilized by a few overseas students to continue their education in Georgia, Kazakhstan, and Kyrgyzstan, among others, being possible mainly through existing tie-ups between these schools and Ukrainian schools. While this system had been in place pre-crisis, albeit not as a governmental initiative, it had two main issues: (1) until recently, it had no official support, and (2) it was highly dependent on the whims of these schools, as well as middlemen, who often exploited these students to earn greater commissions [81,85,86]. There have been allegations of Ukrainian universities and the agents common between the Ukrainian universities and universities in other countries, of not releasing their marksheets and other educational documents, in an effort by the universities to hold on to their students [81]. Some Ukrainian universities have asked their students to come to the still-war torn country and pay their full due fees and collect their documents. However, with the recent announcement of support from the government and the NMC, it is likely that a fair, non-exploitative system can be created. As it stands today, the inclusion of more participating schools is needed, but it represents an ethically gracious and equitable solution to these sufferers of extraordinary events. Foreign medical schools need to roll out detailed transfer pathways and associated policies, specifically for Ukraine-evacuated students, in a timely, transparent manner. This solution is formally supported by the authors of this review, even though it requires significant logistical maneuvering, including governmental efforts from India as well as the recipient country.

Meanwhile, returning students have themselves been searching for options. Education consultants have reported that medical schools in Bangladesh, Nepal, and Kyrgyzstan have received significant interest [106]. Russia has announced that these students may continue their education there, with some universities even offering discounts on tuition and accommodation fees [107]. However, given their status as primary aggressors in this conflict, many students have expressed opposition to this [108]. Given the concerning status of all individuals leaving Ukraine, it may be argued that there exists some degree of moral imperative for high-income countries to incorporate these students into their own medical schools. The counterargument is that these returning students are distinct from Ukrainian refugees, who have been defined by the 1951 Refugee Convention to be ‘someone who is unable or unwilling to return to their country of origin owing to a well-founded fear of being persecuted for reasons of race, religion, nationality, membership of a particular social group, or political opinion’ [109]. For HICs, these Indian-origin students are potentially immigrants, and likely no ethical obligation exists for integrating students. Similar to India, considerable public discontentment may potentially occur in HICs as well if medical school spots were provided to non-Ukrainian immigrants.

Another solution, fully discussed for the first time in this article, is to reintegrate all returning students through a one-time mandated expanded enrolment by 10–20%, at all medical schools in India, both public and private, a capacity expansion size that would avoid significant overburdening. While this solution requires considerable legal support and faces the challenge of fair allotment of medical school spots, it is, however, ethically appropriate. The difficulty would be in deciding on a merit list based on which a round of counseling may be carried out. Creating a new rank list may be done in several ways – (A) using the original NEET-UG percentiles of returning candidates, a method that is fair, equitable, and feasible; (B) using students’ performance in the Ukrainian medical schools, which may have problems related to transcript verification, and difficult logistics of merging different grading schemas into one standardized measure through manual assessment; or (C) using a new norm-referenced examination, testing knowledge of basic sciences, held solely for Ukrainian medical students, which is fair but faces financial and logistical barriers to designing and conducting a national examination.

Each solution to this complex issue carries its limitations. However, given that over six months have passed since the return of the students, additional delays may decrease the utility of this large future workforce and may lead to further unrest. A lack of public clarity and transparency regarding newly proposed regulations related to FMGE and foreign medical schools has led to systemic uncertainty [110]. National advocacy and urgent decision-making are required to ensure the maximum benefit to the currently involved students, future enrollees in foreign medical schools, and the country’s healthcare workforce.

## Conclusions

**Figure 1:**
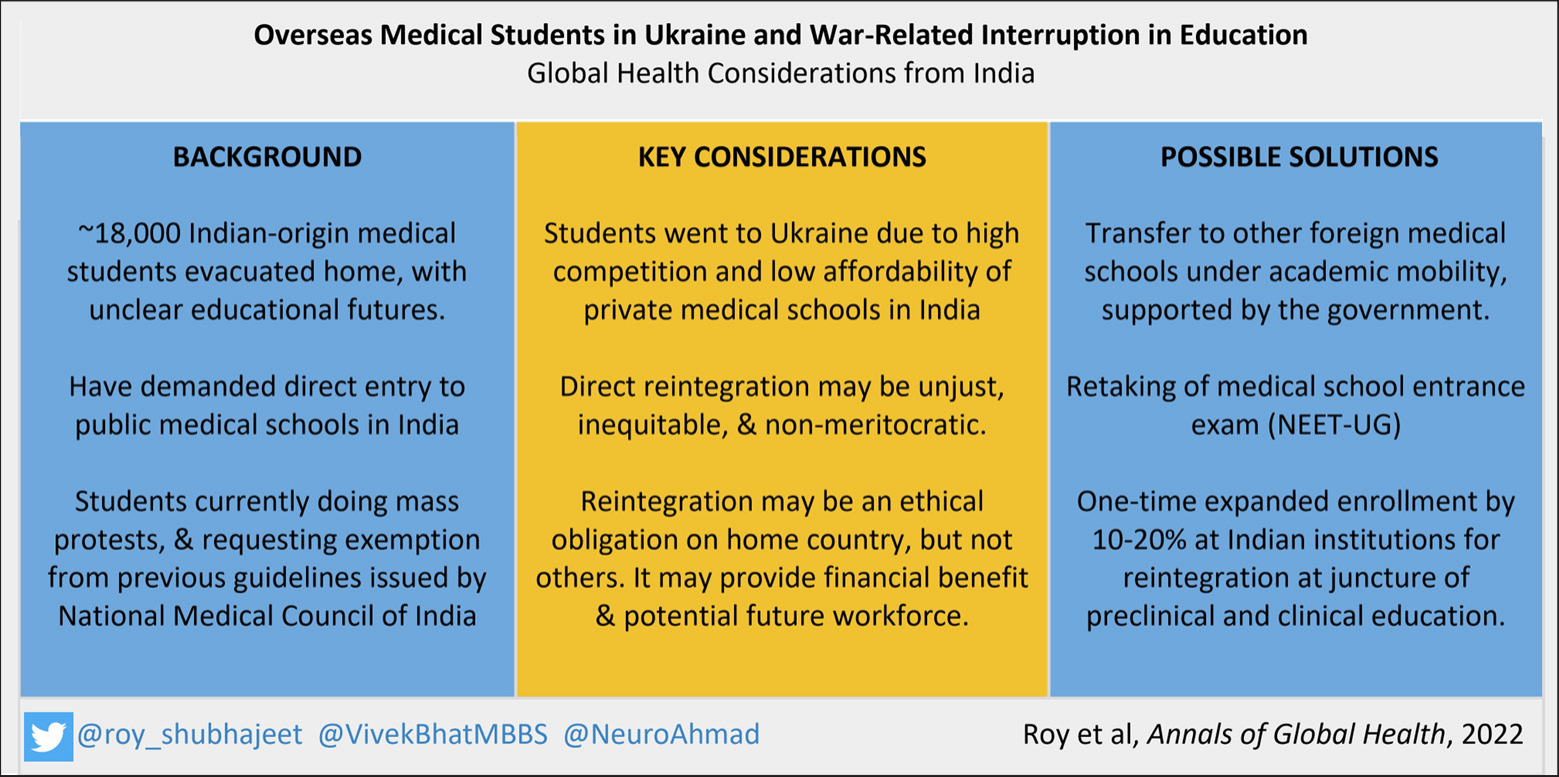
Summary of major aspects related to the Indian-origin medical students who were enrolled in Ukrainian medical schools and had their education interrupted.

Urgent attention is needed to address the educational status of 18,000 Indian medical students, who await clarity following their return from Ukraine more than six months ago (Figure 1). Potential challenges to their reintegration into the Indian medical system include ethical and sociocultural concerns surrounding their meritocracy, as well as significant logistical hurdles. These challenges have been reflected in the less-than-favorable decisions by key legislative and executive authorities. However, given the extraordinary circumstances, the potential economic benefits to their integration, as well as the dire need for trained physicians in India, a measured approach allowing their integration could be of significant value to the country. Potential solutions, for which federal support may help, include academic mobility involving other foreign medical schools; or retaking of the medical school entrance examination by returning students; or their reintegration with credit transfer into Indian medical schools through a one-time enrollment expansion. The latter may be done using their original standardized test percentiles, or performance in Ukrainian medical schools, or new national assessment. While the involvement of all stakeholders and careful consensus-building is warranted, urgent decision-making is critical to prevent resource wastage.
